# Resolution of severe eosinophilic peritonitis in a patient on continuous ambulatory peritoneal dialysis by changing from Dianeal peritoneal dialysis solution to Stay-Safe Balance solution: a case report

**DOI:** 10.1186/s12882-022-02786-8

**Published:** 2022-04-18

**Authors:** Zi Chan, Yun Ho Hui, Sunny Sze Ho Wong

**Affiliations:** Department of Medicine, Renal Unit, United Christian Hospital, 130 Hip Wo Street, Kwun Tong, Kowloon, Hong Kong

**Keywords:** Peritoneal dialysis, allergic reaction, eosinophilic peritonitis, treatment, case report

## Abstract

**Background:**

Eosinophilic peritonitis is a well-known complication at the initiation of peritoneal dialysis. It is often due to an allergic reaction to peritoneal dialysis solution or sometimes to the peritoneal dialysis catheter itself. Most cases are self-limiting with expected spontaneous resolution within a few weeks. Treatment is necessary for severe or persistent cases. However, the optimal treatment has not yet been defined. There have been only a few case reports on the use of anti-histamines and/or steroids for the treatment of eosinophilic peritonitis. We reported a case of severe eosinophilic peritonitis successfully treated by switching the brand of peritoneal dialysis fluid (same glucose concentration). To the best of our knowledge, this is the first reported case employing such a treatment strategy.

**Case presentation:**

An eighty-two-year-old man with end-stage renal failure was started on continuous ambulatory peritoneal dialysis with Dianeal® (Baxter) peritoneal dialysis solution. He developed eosinophilic peritonitis 19 days after initiation of peritoneal dialysis. Infectious causes were ruled out by bacterial, fungal and tuberculosis smears and cultures. In view of the persistent and severe symptoms, we intervened by switching from Dianeal® (Baxter) peritoneal dialysis solution to Stay-Safe Balance® (Fresenius) solution with the same glucose concentration. His eosinophilic peritonitis resolved 5 days after switching the peritoneal dialysis solution.

**Conclusion:**

In severe or persistent cases of eosinophilic peritonitis requiring treatment, other than the use of steroids and anti-histamines, switching to a different brand of peritoneal dialysis solution can be considered.

## Background

Eosinophilic peritonitis is defined as an absolute eosinophil count greater than 100/mm^3^ in the peritoneal dialysis fluid (PDF) effluent or when eosinophils constitute more than 10% of the total white blood cell (WBC) count [1]. It tends to occur early (within 3 months) after initiating peritoneal dialysis (PD) and is attributed to hypersensitivity reactions to PD materials, including PDF or the PD catheter itself [2]. It can also be induced by medications [3, 4]. Occasionally, eosinophilic peritonitis can occur with fungal or parasitic infections, or it can occur during the treatment phase of bacterial peritonitis. Careful clinical history and laboratory tests including culture of the PDF effluent are needed to distinguish infectious from non-infectious causes.

## Case presentation

An eighty-two-year-old man with end-stage renal failure due to polycystic kidney disease was newly started on PD. He was put on intermittent PD two weeks after PD catheter insertion and was later started on continuous ambulatory peritoneal dialysis at one month. His PD regimen consisted of 1.5% 2L Dianeal® (Baxter) PDF three bags exchanged per day. He was admitted to the hospital 19 days later for turbid PDF effluent. The total PDF effluent WBC count was 5643/mm^3^, with 29% neutrophils, 2% lymphocytes, 13% monocytes and 56% eosinophils. He did not have fever or abdominal pain. Intraperitoneal vancomycin and amikacin were started empirically upon admission. Antibiotics were chosen in view of the patient’s known allergies to penicillin, erythromycin and levofloxacin. He had also been taking amlodipine, lisinopril, terazosin, frusemide, ferrous sulphate, pantoprazole, vitamin B complex, vitamin C and methoxy polyethylene glycol epoetin beta subcutaneous injection. There had been no change in medications since PD catheter insertion. The bacterial and fungal cultures of the PDF effluent and a polymerase chain reaction test for *Mycobacterium tuberculosis* complex DNA were all negative. However, the PDF effluent was persistently turbid with a marked decrease in ultrafiltration. Eosinophilic peritonitis due to an allergic reaction was suspected. All intraperitoneal antibiotics were stopped, with oral chlorpheniramine prescribed on day 11 after admission. The PDF effluent remained turbid, with a total WBC count ranging from 5000-6000/mm^3^. We switched the 1.5% 2L Dianeal® (Baxter) PDF to 1.5% 2L Stay Safe Balance® (Fresenius) PDF on day 14 after admission. There was significant improvement in the turbidity of the PDF effluent the next day (total WBC count 1320/mm^3^, eosinophils 72%), and it eventually cleared up on day 19 (total WBC count 40/mm^3^). There was no recurrence of turbid PDF effluent until the latest follow-up at one month after discharge.

## Discussion and Conclusion

Eosinophilic peritonitis due to an allergic reaction is a well-known complication of PD. It ranges from asymptomatic mild turbidity of the PDF effluent, which is sometimes only demonstrated after nocturnal dwelling, to overtly turbid PDF effluent with symptoms. Mild eosinophilic peritonitis is most often self-limiting and resolves spontaneously, while severe cases may persist and require treatment. There is a paucity of data for the optimal treatment of clinically significant eosinophilic peritonitis. There have been a few case reports on the successful treatment of eosinophilic peritonitis with steroids [5–7], anti-histamines [7, 8] and montelukast [9].

We reported a case of severe eosinophilic peritonitis successfully treated by switching the brand of PDF. To the best of our knowledge, this is the first reported case employing such a treatment strategy. There have been reports on allergic reactions to icodextrin solution used in PD, with resolution after switching to standard glucose-based PDF [10, 11]. Some of the earlier cases were probably due to the contamination of some icodextrin batches by peptidoglycans during manufacture [12]. However, there has been no previous report on treatment by switching from one glucose-based PDF to another.

The Stay-Safe Balance PDF consists of two-compartment solution bags, which are allowed to mix at the time of use. It has the advantage of providing a low glucose degradation product solution at neutral pH when compared to the conventional bag system employed by other PDFs. However, there is no consistent evidence on whether the more physiological solution can actually translate into clinical benefits [13, 14]. It is unclear in our case whether the resolution of eosinophilic peritonitis was due to the above-mentioned properties of the Stay-Safe Balance® (Fresenius) PDF or whether it was due to the difference in their excipients or materials used for the plastic container bags. According to the manufacturers, all components of the packaging for Stay-Safe Balance® (Fresenius) solution are made of polyvinyl chloride (PVC)-free Biofine®, whereas Dianeal® (Baxter) solutions are packed in bags made of PVC. We avoided the use of steroids, which are the most reported agents used for the treatment of eosinophilic peritonitis, so that the side effects of steroids could be avoided in this elderly patient. Switching of the brand of PDF represents a reasonable choice for the treatment of severe eosinophilic peritonitis with few potential side effects.

We did not perform any rechallenge test for this patient, as the inflammatory response was severe, and the temporal sequence was compatible with the allergic response to the initial choice of PDF. The patient also had history of allergy to multiple groups of antibiotics. He might have an underlying increased tendency to hypersensitivity reaction. Although the causal relationship between Dianeal® (Baxter) PDF and eosinophilic peritonitis could not be directly proven in this case, their apparent associations merit consideration.

Eosinophilic peritonitis can be caused by an allergic reaction to PDF, drugs or, in some cases, infectious processes, such as fungal peritonitis. After ruling out infectious peritonitis, most cases can be managed conservatively with expected spontaneous resolution. In severe or persistent cases, other than the use of steroids and anti-histamines, switching to a different brand of PDF can be considered.

## Data Availability

The datasets used and/or analysed during the current study are available from the corresponding author upon reasonable request.

## Abbreviations

PD: Peritoneal Dialysis
PDF: Peritoneal Dialysis Fluid
PVC: Polyvinyl Chloride
WBC: White Blood Cell
